# Modulation of the oscillatory mechanics of lung tissue and the oxidative stress response induced by arginase inhibition in a chronic allergic inflammation model

**DOI:** 10.1186/1471-2466-13-52

**Published:** 2013-08-15

**Authors:** Luciana RCRB Aristoteles, Renato F Righetti, Nathalia Montouro Pinheiro, Rosana B Franco, Claudia M Starling, Julie CP da Silva, Patrícia Angeli Pigati, Luciana C Caperuto, Carla M Prado, Marisa Dolhnikoff, Milton A Martins, Edna A Leick, Iolanda FLC Tibério

**Affiliations:** 1 Department of Clinical Medicine, School of Medicine, University of Sao Paulo, 01246-903 São Paulo, SP, Brazil; 2 Department of Clinical Medicine and Pathology, School of Medicine, University of Sao Paulo, 01246-903 São Paulo, SP, Brazil; 3 Department of Biological Sciences, Universidade Federal de São Paulo, Diadema, SP, Brazil; 4 Faculty of Medicine, University of São Paulo, Av. Dr. Arnaldo, 455 - Sala 1210, 01246-903 São Paulo, SP, Brazil

**Keywords:** Lung parenchyma, Arginase, iNOS, Nitric oxide, Guinea-pig, nor-NOHA, Oxidative stress

## Abstract

**Background:**

The importance of the lung parenchyma in the pathophysiology of asthma has previously been demonstrated. Considering that nitric oxide synthases (NOS) and arginases compete for the same substrate, it is worthwhile to elucidate the effects of complex NOS-arginase dysfunction in the pathophysiology of asthma, particularly, related to distal lung tissue. We evaluated the effects of arginase and iNOS inhibition on distal lung mechanics and oxidative stress pathway activation in a model of chronic pulmonary allergic inflammation in guinea pigs.

**Methods:**

Guinea pigs were exposed to repeated ovalbumin inhalations (twice a week for 4 weeks). The animals received 1400 W (an iNOS-specific inhibitor) for 4 days beginning at the last inhalation. Afterwards, the animals were anesthetized and exsanguinated; then, a slice of the distal lung was evaluated by oscillatory mechanics, and an arginase inhibitor (nor-NOHA) or vehicle was infused in a Krebs solution bath. Tissue resistance (Rt) and elastance (Et) were assessed before and after ovalbumin challenge (0.1%), and lung strips were submitted to histopathological studies.

**Results:**

Ovalbumin-exposed animals presented an increase in the maximal Rt and Et responses after antigen challenge (p<0.001), in the number of iNOS positive cells (p<0.001) and in the expression of arginase 2, 8-isoprostane and NF-kB (p<0.001) in distal lung tissue. The 1400 W administration reduced all these responses (p<0.001) in alveolar septa. Ovalbumin-exposed animals that received nor-NOHA had a reduction of Rt, Et after antigen challenge, iNOS positive cells and 8-isoprostane and NF-kB (p<0.001) in lung tissue. The activity of arginase 2 was reduced only in the groups treated with nor-NOHA (p <0.05). There was a reduction of 8-isoprostane expression in OVA-NOR-W compared to OVA-NOR (p<0.001).

**Conclusions:**

In this experimental model, increased arginase content and iNOS-positive cells were associated with the constriction of distal lung parenchyma. This functional alteration may be due to a high expression of 8-isoprostane, which had a procontractile effect. The mechanism involved in this response is likely related to the modulation of NF-kB expression, which contributed to the activation of the arginase and iNOS pathways. The association of both inhibitors potentiated the reduction of 8-isoprostane expression in this animal model.

## Background

Asthma is a disease of both the airways and, as recently addressed, the alveolar parenchyma. Some studies have demonstrated the presence of inflammation and the remodeling of lung parenchyma in asthma, both in humans and in experimental models, showing the relevance of distal lung tissue responses in total pulmonary resistance and the effects of inflammatory mediators in peripheral lung changes [1-9].

Nitric oxide (NO) is an important endogenous modulator of airway and distal lung constriction, generated by a family of NO synthase (NOS) isoforms. Studies in macrophages have indicated that under conditions of low L-arginine availability, iNOS not only produces NO via its oxygenase moiety, but its reductase moiety also synthesizes superoxide anions, leading to the efficient formation of peroxynitrite [10]. Subsequently, isoprostane generation may result from lipid peroxidation induced by peroxynitrite formation. PGF2α represents the main member of the isoprostane family, which members are biochemical markers of oxidative stress and are produced through the peroxidation of arachidonic acid [10-12].

Airway inflammation is accompanied by a marked upregulation of iNOS expression, particularly in airway epithelium [13], that has been associated with the activation of nuclear factor-kB (NF-κB) that play critical roles in inflammation, immunity, cell proliferation, differentiation and survival [14]. Further studies showed that NF-κB is activated in the lung tissue of animal models of allergic airway inflammation [15] and of patients with asthma [16], specifically within airway epithelium, and it is known that NF-κB activity can be affected by reactive oxygen species (ROS) as well as reactive nitrogen species (RNS) [17].

Arginases, which convert L-arginine into L-ornithine and urea, are key enzymes of the urea cycle in the liver (arginase 1) but are also expressed in cells and tissues that lack a complete urea cycle, for example, arginase 2 expression in the lung [18].

Que et al. [19] demonstrated the expression of arginase in the bronchial epithelium and in peribronchial connective tissue fibroblasts. In addition, Meurs et al. [18] showed that arginase appears to modulate the tone of airway smooth muscle and potentiates methacholine-induced airway constriction. Arginase accomplishes this by competing the common substrate L-arginine away from epithelial cNOS to diminish agonist-induced NO production. Arginases and NOS compete for the bioavailability of the same substrate, L-arginine, and are involved indirectly in the regulation of NO synthesis [20].

Several powerful drugs have been used to investigate the role of arginase in the pathophysiology of asthma, including nor-NOHA (Nω-hydroxy-nor-Larginine), which is one of the most potent inhibitors of arginase [21]. Meurs et al. [22], studying *in vitro* tracheal ring of sensitized guinea pigs, demonstrated that treatment with nor-NOHA reduced hyperresponsiveness to methacholine, and this effect was reversed by treatment with L-NAME.

Previously, we had used guinea pigs with chronic allergic inflammation treated with a false substrate for all NOS (L-NAME) and a specific iNOS inhibitor (1400 W) to demonstrate that all NOS constitutive isoforms reduced lung responsiveness and inflammatory response and protected against extracellular matrix remodeling. Moreover, NO derived from iNOS activation contributes to increased lung responsiveness, inflammatory cells recruitment and extracellular matrix remodeling both in airways and distal lung parenchyma [2,3,5-7].

Considering these complex interactions, we hypothesis that, increased arginase activity 2 also plays a key role in the pathophysiology of chronic asthma. In order to clarify this statement we evaluated the expression and activity of arginase 2 as well by treatment with a specific inhibitor of arginase (Nu-hydroxy-or L-arginine: nor-NOHA) attenuates mechanical oscillatory responses and oxidative stress of lung tissue in a distal model chronic allergic pulmonary inflammation.

## Methods

Male guinea pigs received humane care in compliance with the “Guide for the care and use of laboratory animals” (NIH publication 85–23, revised 1985), and experiments described in this study were previously approved by the Institutional Review Board of the University of São Paulo.

### Induction of chronic pulmonary allergic inflammation

**Figure 1:**
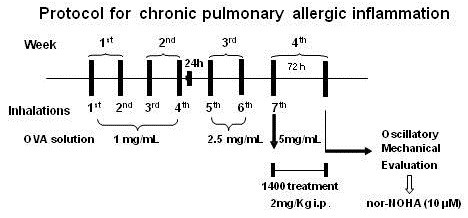
**Timeline of the experimental protocol.** The guinea pigs underwent 7 inhalations (2 per week with 2- to 3-day intervals over 4 weeks) with aerosols of normal saline or ovalbumin solution and increasing doses of antigen. For the 1^st^ through the 4^th^ inhalations, the dose used was 1 mg/mL of ovalbumin (2 weeks). In the 5^th^ and 6^th^ inhalations (3^rd^ week), the animals inhaled 2.5 mg/mL of ovalbumin, and for the 7^th^ inhalation (beginning in the 4^th^ week) 5 mg/mL of antigen was used. Treatment with 1400W started 30 minutes before the 7^th^ inhalation and was given daily until the oscillatory mechanics assessment. The solution of ovalbumin or normal saline was continuously aerosolized for 15 minutes or until respiratory distress occurred. Seventy-two hours after the 7^th^ inhalation, the animals were anesthetized, exsanguinated, and their lungs were removed for oscillatory mechanics measurement. Nor-NOHA (10 μM) was infused in the bath during the evaluation of oscillatory mechanics.

Male Hartley guinea pigs weighing 300–400 g were placed in a plexiglass box (30 × 15 × 20 cm) coupled to an ultrasonic nebulizer (Soniclear, São Paulo, Brazil). A solution of ovalbumin (OVA, Grade V, Sigma Chemical Co., Saint Louis, MO, USA) diluted in 0.9% NaCl (normal saline) was prepared. For four weeks, the animals received seven inhalations of increasing concentrations of OVA (1~5 mg/mL) to counteract tolerance (Figure 1). Control animals received aerosolized normal saline (SAL group). The solution was continuously aerosolized into the environment until respiratory distress occurred, as previously described [4,6,23]. The observer who made the decision to withdraw the guinea pig from the inhalation box was blinded to the treatment status of the animal.

### 1400W administration

1400 W (Tocris Bioscience, Ellisville, MO, USA) (a specific and highly selective iNOS-inhibitor) was given *ip* for four days at 2 mg/kg/animal/day (OVA-W group), beginning 30 minutes before the 7^th^ inhalation of either ovalbumin or normal saline. This approach was chosen based on previous data [6,24,25]. As high doses of 1400 W (50 mg/kg *ip*) can cause toxic effects, we chose a lower dose with previously demonstrated proven therapeutic efficacy in experimental models [7,25].

### nor-NOHA administration

Nor-NOHA (Nω-hydroxy-nor-Larginine, Caymam Chemical, Ellsworth Road Ann Arbor, MI, USA) is considered one of the most potent inhibitors of arginase, as previously described [22]. At the beginning of the protocol nor-NOHA (10 μM) was added to the Krebs bath and after 40 minutes we performed OVA (0.1%) challenge. Afterwards, mechanical evaluation was done.

### Experimental groups

The animals were randomly divided into the following experimental groups (n = 6 for each group):

a) Inhalations with sterile saline 0.9% (SAL group);

b) Inhalations with ovalbumin solution (OVA group);

c) Inhalations with ovalbumin solution and treatment with nor-NOHA in the bath (OVA-NOR group);

d) Inhalations with ovalbumin and treatment with 1400 W (*ip*) (OVA-W group);

e) Inhalations with ovalbumin solution and 1400 W (*ip*) and nor-NOHA in the bath (OVA-NOR-W group).

### Oscillatory mechanics measurements

Seventy-two hours after the 7^th^ inhalation, the animals were anesthetized with thiopental (50 mg/Kg *(ip*), tracheostomized, and exsanguinated after thoracotomy. Heart and lungs were excised *en bloc*, and the lungs were infused with Krebs solution (in mM: NaCl, 118; KCl, 4.5; NaHCO_3_, 25.5; CaCl_2_, 2.5; MgSO_4_, 1.2; KH_2_PO_4_, 1.2; glucose 10; all from Sigma Chemical). Sagittal slices of both lungs (defined herein as total lung) were covered with *Optimum Cutting Temperature* (OCT) compound (Reichert-Jung, Heidelberg, Germany) and cooled in liquid nitrogen for subsequent histopathological studies. Subpleural parenchyma strips of the lower lobes (10 mm × 2 mm × 2 mm) were cut, and the resting length (Lr) and wet weight (W0) of each strip were measured. Metal clips were glued to either end of the tissue strips with cyanoacrylate. Steel wires (0.5-mm diameter) were attached to the clips; one side was connected to a force transducer (model 404A; Cambridge Technologies, Ontario, Canada), and the other side was connected to a servo-controlled lever arm (model 300B; Cambridge Technologies). The lever arm was capable of peak-to-peak length excursions of 8 mm and length resolutions of 1 μm. It was connected to a function generator (model 3030; BK Precision, Chicago, IL, USA), which controlled the frequency, amplitude, and waveform of the oscillation.

The resting tension (T) was set by the movement of a screw thumb wheel system, which effected slow vertical displacements of the force transducer. Length and force signals were converted from analog to digital with an analog-to-digital converter and recorded by a compatible computer. The strips were preconditioned three times by slowly cycling tension from 0 to 2 g. After the third cycle, they were fixed at 1 g [8] and maintained in an organ bath containing aerated *Krebs* solution (95% of O_2_, 5% of CO_2_), which was changed every 15 minutes for 50 min to allow stress relaxation. The frequency of oscillation was 1 Hz and the amplitude was 2.5% Lr [8]. Baseline measurements of tissue resistance (Rt) and elastance (Et) were obtained for 5 min, and then the maximal responses of R and E were collected 10 min after a specific antigen challenge with OVA (0.1%) in the bath [26]. The R and E were estimated by the recursive least-squares algorithm to the equation of motion [27].

(1) $$T=E\Delta l+R\left(\Delta l/\Delta t\right)+K$$

where T is tension, l is length, Δl/Δt is the length change per unit of time, and K is a constant reflecting resting tension. The results were standardized for strip size. The unstressed cross-sectional area (*A*_0_) of the strip was obtained from the formula:

(2) $$A0\left({cm}^{2}\right)=W0/\left(p\;x\;\mathrm{Lr}\right)$$

where p is the mass density of the tissue taken as 1.06 g/cm^3^, W0 is the wet weight in grams, and *L*_r_ is the resting length in centimeters. The values of R and E were multiplied by *L*_r_/*A*_0_. We analyzed the percentage of increase in resistance (%R) and elastance (%E) in relation to the baseline [8]. Afterwards, the strips were fixed in 10% formalin for 48 (hours) and embedded in paraffin for histological analysis.

### Morphometric analysis

The homogeneity of the strip samples was assured by measuring the fractional area of tissue constituents with the point-counting method [3,28,29] using a 100-point grid with a known area (62,500 μm at 400× magnification) attached to the ocular of the microscope. We measured the fractional area of bronchial wall (BW), blood vessel wall (BVW), and alveolar wall (AW) as the number of points that fell in either BW, BVW or AW divided by the total number of points that fell in the strip tissue. Measurements were performed in 10 fields per slide at 400× magnification. We have calculated the mean values for each animal. The morphometric analysis was performed by two authors blinded to the experimental groups. The variability of these measurements observed was 1% (coefficient of variation).

### Measurement of iNOS positive cells

The total lung slices covered with OCT and cooled in liquid nitrogen were used for the detection of iNOS positive cells. The cryostat sections (Leica CM1850; Leica, Nosfloch, Germany) were mounted on glass slides pre-coated with aminopropyltriethoxysilane (Sigma Chemical Co.) and fixed in chloroform-acetone (Merck, Rio de Janeiro, Brazil) vol/vol for 10 minutes at room temperature. Immunohistochemistry analysis was performed as previously described [6]. The sections were then incubated for 30 minutes at room temperature with a blocking solution containing normal mouse serum (Dako Corp., Carpinteria, CA, USA).

Monoclonal antisera raised in mouse against iNOS (IgG2a - iNOS/NOS Type II - N32020 - BD Transduction Laboratories, San Diego, CA, USA) [6,7,30] were used as the primary antisera (incubation overnight at room temperature, 1:5 dilution in Tris buffer). After three 5-minute washes in tris-buffered saline (TBS), the sections were incubated with a secondary antibody (LSAB+AP Link Universal, Dako Corp.) for 30 minutes at 37°C in a humid chamber. The slides were given three more 5-minute washes in TBS and were coverslipped with pre-diluted (for 30 minutes) alkaline phosphatase (LSAB + AP - Streptavidin AP - Dako Corp.). This was followed by incubation with the substrate Fast Red TR (Sigma Chemical Co.) for 6 minutes and light hematoxylin counterstaining for 1 minute. Ten fields were analyzed per lung at a magnification of 1000× in an optical microscope (10^4^ μm^2^). The iNOS positive cells were expressed as number of cells/10^4^ μm^2^[6,7].

### Evaluation of NF-kB expression

The immunohistochemistry analysis was carried out using biotin-streptavidin peroxidase. Histological sections 3 μm thick were made on silanized slides (3-Aminopropyl-trietoxy-silane-Sigma) and the protocol described below was followed. The slides were dewaxed, hydrated and the endogenous peroxidase was blocked with hydrogen peroxide (3% H_2_O_2_) 10V 7 times for 5 minutes each, after which the slides were washed with water and PBS. Antigen retrieval was performed in a pressure cooker (Pascal) for 1 minute at 125°C with citrate buffer pH: 6.0. After blocking, the primary NF-kB antibody (cod. SC-109, Santa Cruz Biotechnology, USA, CA), was diluted in BSA 1:50, applied to the slides and incubated overnight. The slides were then washed in PBS and incubated with secondary antibody (1 hour) and complex (30 min) by ABCKit Vectastain (Vector Elite PK-6101 (anti-Rabbit) in an oven at 37°C. After this step, the slides were washed in PBS and the antibody staining visualized through the addition of 3.3 chromogen diaminobenzidine (DAB - Dako K 3466). The slides were washed thoroughly with tap water and counter-stained with Harris hematoxylin (Merck, Darmstadt, Germany). The slides were then washed in water, dehydrated, cleared and mounted with Entellan resin (Merck, Darmstadt, Germany). The NF-kB expression was evaluated under ×400 magnification with an Image-Pro Plus 4.5 v Image Analysis System [6,7], and the results were obtained as a relationship between the quantity of NF-kB expression in a specific frame and the total area of the frame expressed as a percentage.

### Evaluation of PGF2α expression

Immunohistochemical staining was performed using an antibody against anti-8-iso-PGF2α (Oxford Biomedical Research, Rochester Hills, MI, USA) at a 1:500 dilution. The sections were deparaffinized and washed 7 times for 5 minutes with H_2_O_2_10V 3% to inhibit endogenous peroxidase activity. After washes in PBS and water, the antigen retrieval was performed with trypsin for 20 minutes. Afterwards, 3 washes in PBS were performed for 3 minutes each. The sections were incubated with anti-8-epi-PGF2 diluted in BSA overnight. After washes in PBS, ABCKit Vectastain (Vector Elite PK-6105, Burlingame, CA) was used as the secondary antibody and 3,3 Diaminobenzidine (DAB) (Sigma Chemical Co) was used as the chromogen. The sections were counterstained with Harris hematoxylin (Merck) [31]. The PGF2α content was evaluated as described in the section: Evaluation of NF-kB expression.

### Arginase 2 expression

The arginase 2 antibody was evaluated with biotin-streptavidin peroxidase. Histological sections 3 μm thick were made on silanized (3-Aminopropyl-trietoxy-silane-Sigma) slides. The sections were deparaffinized and hydrated, and the endogenous peroxidase was then blocked with 3% hydrogen peroxide (H_2_O_2_) 7 times for 5 minutes each, followed by washes with water and PBS. After blocking, a primary antibody against arginase 2 (cod. 18357, Santa Cruz Biotechnology, USA, CA) was diluted in BSA at 1:120 and incubated on the slides overnight. The sections were then washed in PBS and incubated with the secondary antibody (1 hour) and complex (30 min) using ABCKit Vectastain Vector Elite PK-6105 (anti-Goat) in a 37 degree oven. After this step, the sections were washed in PBS and visualized with 3.3 chromogen diaminobenzidine (DAB - Dako K 3466). The sections were then washed thoroughly with tap water and counter-stained with Harris hematoxylin (Merck, Darmstadt, Germany). Finally, the sections were washed in water, dehydrated, cleared and mounted with Entellan resin for microscopy (Merck, Darmstadt, Germany). The arginase content was evaluated as described in the section: Evaluation of NF-kB expression.

### Arginase 2 activity

After extraction, samples were immediately frozen in liquid nitrogen and stored at −80°C until homogenization. Subpleural parenchyma strips were homogenized in an extraction buffer (20 mM Tris (pH 7.4), 2 uM phenylmethanesulfonylfluoride (PMSF)) with a Polytron PTA 20S generator (model PT 10/35, Brinkmann Instruments, Inc., Westbury, NY, USA) operated at maximum speed for 30 sec. The extracts were centrifuged at 15,000 g, 4°C, for 30 min to remove insoluble material. Protein concentrations of the supernatants were determined by the Bradford assay. QuantiChrom™ Arginase Assay Kit (DARG-200, Bioassay Systems, Hayward, CA, USA) was used to assess the activity of arginase. In short, 40 μl of homogenate was plated into a 96-well plate and 10 μl of 5× substrate buffer (4 vol of Arginine Buffer and 1 vol of the Mn Solution, supplied in the kit) subsequently added. The controls consisted of 40 μl of homogenate without substrate buffer 5×. The plate was incubated at 37°C for 2 hours. To stop the reaction, 200 μl of urea reagent, supplied in the kit, was added to each well and also 10 μl of 5× substrate buffer to control samples. Plate was incubated for 60 minutes at room temperature. The reading was performed at 430 nm and the activity of arginase 2 was calculated by measuring the reaction rate. One unit (U) of arginase converts 1 umol of L-arginine to ornithine and urea per minute at pH 9.5 and 37°C. The specific activity was then expressed in terms of activity per milligram of protein (mU/mg).

### Data analysis

Values were expressed as medians and percentiles and graphics were performed as “Box plot”. The data were examined using *Kruskal-Wallis* non-parametric analysis of variance. Multiple comparisons were made using *Dunn’s* test. A *p* value < 0.05 was considered significant [32]. We also obtained a Spearman correlation coefficient (R) to assess the associations of the Rt scores with the NF-kB, iNOS, isoprostane, PGF2α and arginase 2 expression. A *p* value < 0.05 was considered significant [32].

## Results

### Values were expressed as medians and percentiles [Median (25-75%)]

#### Inhibition of iNOS or Arginase 2 attenuates oscillatory mechanics in sensitized animals

**Figure 2:**
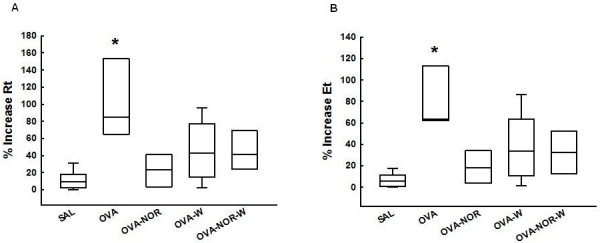
**“Box-plots” of the percentage of increase in tissue resistance (Rt%) (A) and elastance (Et%) of the five experimental groups (B).** *p<0.001 compared to SAL, OVA-NOR, OVA-W, and OVA-NOR-W groups.

The ovalbumin-exposed animals (OVA group) presented an increase in the %Rt [85.0 (69.6-144.0)] and %Et [63.6 (62.1-111.8)] after OVA challenge relative to the saline-exposed ones (SAL group) (%Rt: [9.0 (3.5-18.0)] and %Et: [5.7 (1.3-11.1)] (p<0.001 for all comparisons). In the sensitized animals, the nor-NOHA group (OVA-NOR group) had lower %Rt [23.2 (4.5-35.8)] and %Et [18.2 (4.8-25.7)] than the ovalbumin-exposed (OVA group) or the vehicle-treated animals (p<0.001). The 1400 W administration in ovalbumin-exposed animals (OVA-W group) reduced the %Rt [43.1 (17.0-70.8)] and %Et [33.8 (11.6-57.1)] compared to ovalbumin-exposed (OVA group) and vehicle-treated animals (p<0.001). In sensitized GP, nor-NOHA and 1400 W administration in ovalbumin-exposed animals (OVA-NOR-W group) reduced the %Rt [41.0 (31.5-67.0)] and %Et [32.4 (16.4-50.9)] below values observed in the OVA group (p<0.001). However, there were no differences between the OVA-NOR and OVA-NOR-W groups (Figure 2 A and B).

#### Inhibition of iNOS or Arginase 2 attenuates arginase 2 expression and activity, the number of iNOS positive cells and NF-kB expression

**Figure 3:**
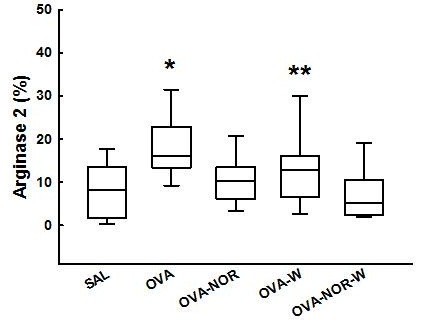
**“Box-plots” of arginase 2 expression (%) in the alveolar septa of the five experimental groups.** *p<0.001 compared to SAL, OVA-NOR, OVA-W, and OVA-NOR-W groups. **p<0.001 compared to OVA-NOR-W group.

There was an increase in the arginase 2 expression (Figure 3) in the ovalbumin-exposed animals (OVA group) compared to the saline-exposed ones (SAL group) [16.0 (13.7-22.7)] and [8.2 (1.8-13.4) %, respectively, p<0.001]. The ovalbumin-exposed animals that received nor-NOHA (OVA-NOR) [10.2 (6.2-13.4) %] and the ovalbumin-exposed animals that received 1400W treatment and nor-NOHA (OVA-NOR-W) [5.1 (2.4-10.3) %] had lower arginase expression than the ovalbumin-exposed animals (OVA group) (p<0.001). The ovalbumin-exposed animals that received 1400W treatment and nor-NOHA (OVA-NOR-W group) had lower arginase expression in lung tissue than the OVA-W animals [12.8 (6.7-16.0) %, p<0.001]. However, there were no differences between the OVA-NOR and OVA-NOR-W groups.

**Figure 4:**
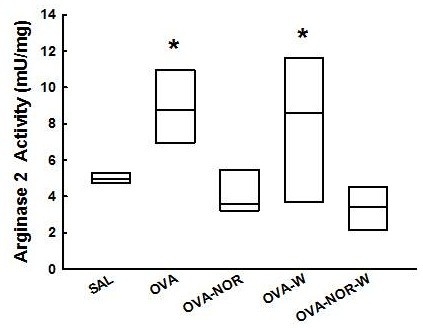
**“Box-plots” of arginase 2 activity in the alveolar septa of the five experimental groups.** *p<0.05 compared to SAL, OVA-NOR and OVA-NOR-W groups. There were no differences between OVA and OVA-W groups.

There was an increase in arginase 2 activity (Figure 4) in ovalbumin-exposed animals (OVA group) relative to the saline-exposed animals (SAL group) [8.8 (7.0-10.8) mU/mg] and [4.9 (4.7-5.2) mU/mg] respectively, p<0.001). The ovalbumin-exposed animals that received nor-NOHA (OVA-NOR) had lower arginase 2 activity [3.6 (3.3-4.9) mU/mg] than the ovalbumin exposed animals (OVA group) (p<0.05). The ovalbumin-exposed animals that received both 1400W treatment and nor-NOHA (OVA-NOR-W group) had lower arginase expression in lung tissue than the OVA-W animals [3.4 (2.4-4.3) mU/mg] and [8.6 (4.4-11.5) mU/mg, respectively p<0.05]. Nevertheless, there were no differences between the OVA-NOR and OVA-NOR-W groups.

**Figure 5:**
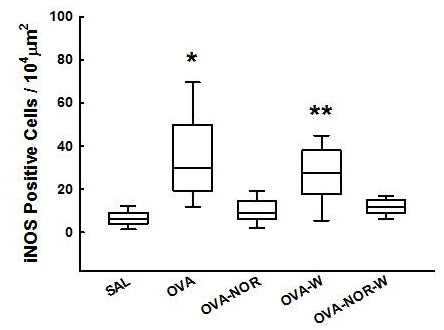
**“Box-plots” of iNOS-positive cells in the alveolar septa of the five experimental groups.** *p<0.001 compared to the SAL, OVA-NOR, and OVA-NOR-W groups. **p<0.001 compared to OVA-NOR-W group.

The ovalbumin-exposed animals (OVA group) showed an increase in the number of iNOS positive cells (Figure 5) in the alveolar septum [29.8 (19.3-49.2) cells/10^4^ μm^2^] compared to the animals exposed to saline (SAL group) [6.4 (3.8-9.2) cells/10^4^ μm^2^]. The ovalbumin-exposed animals that received nor-NOHA (OVA-NOR) showed a lower number of iNOS positive cells [9.1 (6.1-14.7) cells/10^4^μm^2^] than the ovalbumin exposed ones (OVA group) (p<0.001. The ovalbumin-exposed animals that received 1400 W and nor-NOHA (OVA-NOR-W group) had a lower amount of iNOS positive cells in their lung tissue than the OVA-W animals [11.7 (9.0-15.1)] and [27.5 (17.8-37.8) cells/10^4^ μm^2^, respectively p<0.001]. However, there were no differences between the OVA-NOR and OVA-NOR-W groups.

**Figure 6:**
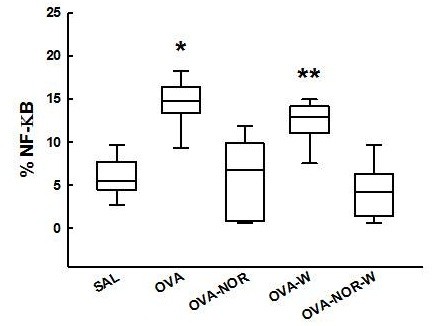
**“Box-plots” of NF-kB expression (%) in the alveolar septa of the five experimental groups.** *p<0.05 compared to SAL, OVA-NOR, OVA-W and OVA-NOR-W groups. **p<0.001 compared to SAL, OVA-NOR and OVA-NOR-W groups.

Ovalbumin-exposed animals (OVA group) showed an increase in NF-kB expression (Figure 6) in the alveolar septum [14.8 (13.3-16.3) %] compared to the animals exposed to saline (SAL group) [5.5 (4.5-7.5) %]. The ovalbumin-exposed animals that received nor-NOHA (OVA-NOR) showed less NF-kB expression [6.8 (0.9-9.8) %] than the ovalbumin exposed animals (OVA group) (p<0.001). Treatment with 1400 W diminished the NF-kB expression in the sensitized animals (OVA-W group) compared to the ovalbumin exposed ones (p<0.001). The ovalbumin-exposed animals that received both 1400 W and nor-NOHA (OVA-NOR-W group) showed a lower expression of NF-kB in lung tissue than the OVA-W animals [4.2 (1.7-6.3) %] and [13.0 (11.1-14.1) %, respectively, p<0.001]. However, there were no differences between the OVA-NOR and OVA-NOR-W groups.

#### The association of iNOS and Arginase 2 inhibition potentates the reduction of isoprostane PGF2α expression

**Figure 7:**
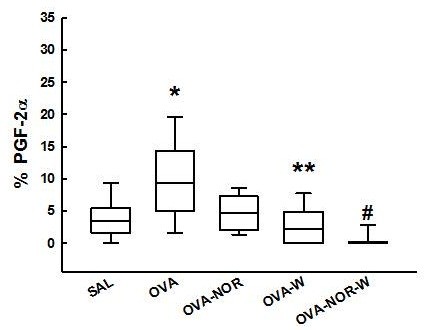
**“Box-plots” of PGF2α expression (%) in the alveolar septa of the five experimental groups.** *p<0.001 compared to SAL, OVA-NOR, OVA-W and OVA-NOR-W groups. **p<0.001 compared to OVA-NOR group. ^#^p<0.001 compared to SAL and OVA-NOR.

We observed an increased expression of PGF2α (Figure 7) in the alveolar septum of guinea pigs exposed to ovalbumin (OVA group) [9.4 (5.0-14.3) %] compared with those exposed only to saline (SAL group) [3.5 (1.6-5.5) %], to that ovalbumin-exposed animals that received nor-NOHA (OVA-NOR group) [4.7 (2.2-7.1) %] to that ones that received 1400 W (OVA-W group) [2.1 (0.0-4.8) %] and to that ovalbumin-exposed animals that received 1400 W and nor-NOHA (OVA-NOR-W group) [0.1 (0.0-0.2) %] (p<0.001 for all comparisons). The ovalbumin-exposed animals that received 1400 W (OVA-W) showed a lower expression of PGF2α in the alveolar septum than the OVA-NOR animals. There was a significant reduction of PGF2α in the alveolar septum in the OVA-NOR-W group compared to the SAL, OVA-NOR and OVA-W groups (p<0.001).

### Correlation of arginase 2, Isoprostane PGF2α, iNOS, NF-kB with distal lung mechanics

In order to better understand all the results and the mechanisms involved, we also performed an analysis of correlation among the functional parameters and the histopathologycal findings. We observed a positive and correlation between Rt with NF-kB expression (R=0.5 and p<0.002); with iNOS positive cells (R=0.762 and p<0.001); Isoprostane PGF2α (R=0.375 and p<0.05) and arginase 2 expression (R=0.569 and p<0.001) (Table 1). In addition, we observed a positive and correlation between NF-kB expression with iNOS positive cells (R=0.573 and p<0.01) and arginase 2 expression (R=0.548 and p<0.01) (Table 1).

**Table 1:** ***Spearman *correlation: arginase 2, Isoprostane PGF2α, iNOS, NF-kB, %Et and %Rt**

		**Isoprostane**	**iNOS**	**NF-kB**	**%Rt**	**%Et**
**Arginase 2**	R	0.508	0.519	0.548	0.569	0.0847
	p	0.00367	0.00293	0.00150	0.000909	0.648
**Isoprostane PGF2α**	R		0.121	0.365	0.375	0.0434
	p		0.512	0.0433	0.0379	0.814
**iNOS**	R			0.573	0.762	0.220
	p			0.000810	0.000000200	0.232
**NF-kB**	R				0.533	0.443
	p				0.00216	0.0129

R = Correlation Coefficient.

p = Value.

### Descriptive analysis

**Figure 8:**
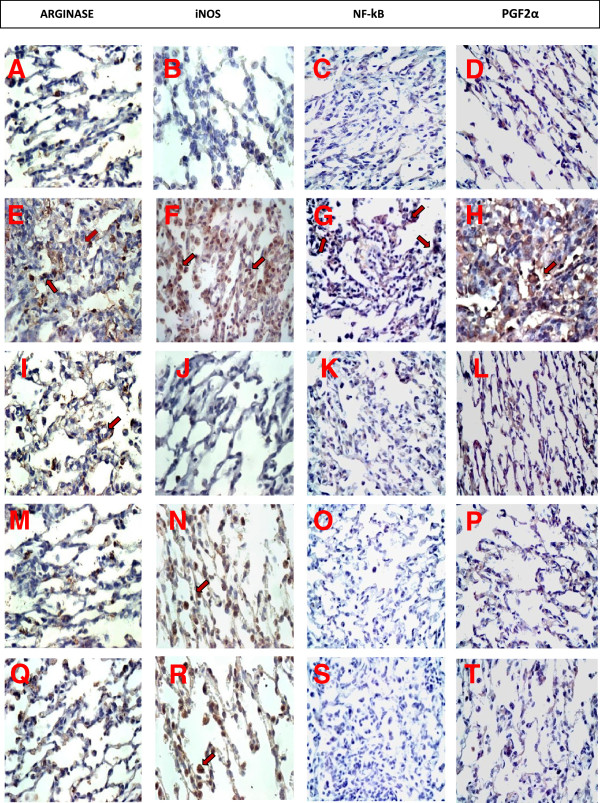
**Guinea pig lung tissue samples obtained from controls (panels A, B, C and D), OVA-exposed and vehicle-treated animals (panels E, F, G and H), OVA-exposed and nor-NOHA treated animals (panels I, J, K and L), OVA-exposed and 1400 W treated animals (panels M, N, O and P), and OVA-exposed and nor-NOHA and 1400 W treated animals (panels Q, R, S and T).** Immunohistochemistry analysis for arginase (panels **A**, **E**, **I**, **M** and **Q** - ×400), iNOS positive cells (panels **B**, **F**, **J**, **N** and **R** - ×1000), NF-kB (panels **C**, **G**, **K**, **O** and **S** – ×400), and PGF2α (panels **D**, **H**, **L**, **P** and **T** - ×400). The control group showed low amounts of iNOS positive cells, NF-kB, arginase and PGF2α in alveolar tissue sections. In contrast, the distal lung parenchyma of OVA-exposed and vehicle-treated animals showed an increase in the amount of arginase, iNOS-positive cells, NF-kB and PGF2α. The 1400 W and nor-NOR treatments in ovalbumin-exposed animals reduced all these parameters compared to the OVA group. Both treatments in conjunction contributed to a greater PGF2α reduction.

The control group (SAL group) showed low amounts of arginase 2, iNOS positive cells, NF-kB and PGF2α content in alveolar tissue sections, coincident with the maintenance of the histoarchitecture of the alveolar septa. In contrast, the distal lung parenchyma of OVA-exposed and vehicle-treated animals showed an increase in iNOS positive cells and in the amount of arginase, NF-kB and PGF2α. The 1400 W and NOR-NOHA treatments in ovalbumin-exposed animals (OVA-NOR-W group) reduced all these parameters below the amounts detected in the OVA group. The association of both treatments caused larger reductions than either treatment alone in isoprostane PGF2α expression (Figure 8).

## Discussion

Previously, we have already shown that the mainly cells present in airway walls of this asthma model are eosinophils, CD4+ lymphocytes, iNOS and nNOS positive cells [4,6,24,28]. In addition, we had demonstrated in distal lung of this animal asthma model that eosinophil density, iNOS positive cells and isoprostane PGF2α were increased due to the effect of the sensitization process. These histopathologycal alterations were correlated with distal lung mechanics responses either after acethylcholine or antigen challenge [2,3,28].

In the present study, we demonstrated that chronic distal lung inflammation was associated with an increase in arginase content and iNOS positive cells. These results were associated with constriction of the distal lung parenchyma. Drazen, Schneider [33] showed that ultrathin guinea-pig lung strips, which contained no conducting airways or blood vessels, contracted after histamine or carbachol challenge. These investigators concluded that alveolar interstitial cells and/or alveolar duct smooth muscle were responsible for the response*.* Although in the present study the strips were represented by more than 90% alveolar walls (Table 2) it was not possible to exclude a constrictor effect of some distal airways and vessels.

**Table 2:** Morphometric determination of distal proportions of alveolar septa, airways, and vessels

**Groups**	**% of alveolar septa**	**% of vessels**	**% of airways**
SAL	90.7 (90.3-90.9)	9.3 (8.7-10.7)	0.8 (0.6-2.0)
OVA	90.3 (89.6-91.4)	8.6 (7.6-9.1)	1.1 (0.9-1.4)
OVA-NOR	91.5 (92.5-91.5)	7.1 (5.7-7.7)	0.6 (0.4-0.8)
OVA-W	90.2 (90.1-90.4)	10.1 (9.4-10.6)	0.7 (0.4-1.2)
OVA-N0R-W	93.1 (90.1-93.8)	5.6 (6.4-5.7)	0.4 (0.6-0.4)

There were no significant differences between the proportions of alveolar septa, airways, and vessels among the five experimental groups.

Median (25-75%).

The increased iNOS expression leads to activation of the oxidative stress pathway and formation of PGF2α, which had a procontractile effect. In addition, we showed that the mechanism involved in the activation of arginase and the iNOS pathways may be related to the modulation of NF-kB expression. Finally, we demonstrated that the association of both inhibitors potentiated the reduction of isoprostane PGF2α expression in this animal model.

The efficacy of these treatment protocols was demonstrated by the significant reduction of the number of iNOS-positive cells and arginase content in the lung tissue of animals exposed to ovalbumin and treated with 1400 W and nor-NOHA, respectively. We confirm previous studies showing that 1400W has *in vivo* selectivity for iNOS [24] and that nor-NOHA is a potent and highly selective inhibitor of arginase [34]. In fact, the nor-NOHA inhibition used is specific for arginase 2. Then the mechanism involved in the modulation of iNOS pathway by arginase is not related to nor-NOHA competition.

Considering the mechanisms involved in an allergic inflammatory microenvironment pro-inflammatory cytokines, like IL-4, IL-1β, and tumour necrosis factor α (TNF-α) and oxidative stress might *upregulate* the production of iNOS-derived NO through activation of transcription factors, such as signal transcription 1 (STAT1), STAT6 and nuclear factor kB (NF-kB) [35]. In our study, arginase 2 blockade leads to decreased NF-KB expression. The treatment with nor-NOHA also leads to a reduction of iNOS expression. This effect could be explained by the fact that iNOS expression is NF-KB dependent.

However, we observed differences in the intensity of the effects of 1400 W and nor-NOHA on the number of iNOS positive cells. We considered that it may be dependent on the doses and / or the differences between the protocols of treatments used.

It is noteworthy that, we demonstrated for the first time in an animal model of chronic allergic inflammation, an increase in arginase 2 expression in distal lung parenchyma. A number of studies have reported an increase in arginase activity in the airways of both acute and chronic guinea pig models of allergic asthma [18,20]. Increased arginase 2 activity and/or expression have also been demonstrated in other animal models using different antigens, species and strains [36].

Some studies also hypothesized that endogenous arginase activity could affect both the proximal and distal airways. Using a perfused guinea pig tracheal tube preparation, Meurs et al. [18] demonstrated that the potent and highly specific arginase inhibitor nω-hydroxy-nor-l arginine (nor-NOHA) caused a concentration-dependent inhibition of methacholine-induced airway constriction, which was reversed by L-NAME. This result shows that arginase activity in the airways is also involved in the modulation of airway responsiveness by limiting cNOS-derived bronchodilating NO production.

Maarsingh et al. [37] showed that in sensitized animals there was an increase in arginase activity, which was completely prevented by the administration of ABH (an arginase inhibitor). So do we, using a different arginase inhibitor (nor-NOHA). Nonetheless, Maarsingh et al. [37] evaluated the proximal airways while our study was performed in the distal lung parenchyma.

Using a guinea-pig model of allergic asthma, De Boer et al. [38] investigated the role of nitric oxide in allergen induced airway hyperresponsiveness after the early asthmatic reaction by examining the effects of the NO synthase inhibitor L-NAME on the responsiveness to methacholine and histamine of isolated perfused tracheae from unchallenged and sensitized animals. The authors also demonstrated that a NO deficiency contributes to allergen-induced airway hyperresponsiveness after the early airway responsiveness.

In asthmatic airways, the expression of iNOS is markedly increased by proinflammatory cytokines such as TNF-α and IFN-ɣ in inflammatory cells and in the airway epithelium [39,40]. Prado et al. [7] also found a reduction in iNOS and nNOS positive cells after treatment with this specific iNOS inhibitor (1400 W) and L-NAME, respectively. Few authors, however, have assessed the effects of inhibition of nitric oxide synthases in modulating the inflammatory and functional responses that occur in the lung parenchyma in the chronic inflammatory response.

In this regard, Angeli et al. [3] analyzed the oscillatory mechanical responses of distal lung tissue in guinea pigs with chronic pulmonary inflammation. The authors observed that sensitized animals treated with L-NAME had a reduction in both lung tissue resistance and elastance after antigen challenge. Starling et al. [2], assessing the distal lung functional changes induced by chronic inflammation, found that treatment with 1400 W decreased the response of both elastance and resistance in animals exposed to ovalbumin. In conjunction, these studies showed that nitric oxide contributes to the constriction of the pulmonary parenchyma in this experimental model.

Considering that L-arginine is the common substrate for both nitric oxide synthases and for the arginase enzyme, it is possible that blocking one of these enzymatic pathways could affect the other one through competition for the same substrate [20,22]. These enzymes convert L-arginine into L-ornithine and urea and are the key enzymes of the urea cycle in the liver (arginase 1) but are also expressed in cells and tissues that lack a complete urea cycle, e.g., arginase 2 expression in the lung [21]. Arginases are involved in cell growth and tissue repair via the increased production of L-ornithine, a precursor of polyamines and proline [21].

Que et al. [41] demonstrated the expression of arginase in the bronchial epithelium and in peribronchial connective tissue fibroblasts. In addition, Meurs et al. [22] showed that arginase appears to modulate the tone of the airway smooth muscle and potentiates methacholine-induced airway constriction. Arginase accomplishes these actions by forcing the common substrate L-arginine away from epithelial cNOS to diminish the agonist induced production of NO. Arginases and NOS compete for the bioavailability of the same substrate, L-arginine, and are involved indirectly in the regulation of NO synthesis [18,21].

Corroborating this idea, Morris et al. [42] showed that there is a reduction in the levels of plasma arginine in asthmatic patients compared with patients without asthma but with increased serum arginase activity. Together, these results suggest that increased arginase activity in asthma may be a contributing factor to the decrease in the circulating levels of Larginine and the consequent NO deficiency. Thus, blocking NO production could be a tool to study the indirect involvement of arginase in various pathophysiological processes [22,43].

Several powerful drugs have been used to investigate the role of arginases in the pathophysiology of asthma, including nor-NOHA (Nω-hydroxy-nor-L-arginine), which is one of the most potent inhibitors of arginase [21]. Meurs et al. [22], studying in vitro tracheal ring-sensitized guinea pigs, demonstrated that treatment with nor-NOHA reduced the hyperresponsiveness to methacholine, and this effect was reversed by treatment with L-NAME.

In the present study, we evaluated the effects of arginase 2 inhibition on functional alterations of distal lung parenchyma. We demonstrated that nor-NOHA decreased both the maximal increase in tissue resistance and elastance after ovalbumin challenge in sensitized animals, suggesting that arginase 2 contributes to the modulation of lung parenchyma specific hyperresponsiveness in this experimental model.

One possible explanation for these results may be related to a decrease in the production of bronchodilating NO, presumably through competition with cNOS for the common substrate, L-arginine, as previously shown in other studies discussed above [18]. In the lungs, the constitutive NOS (cNOS) isozymes are mainly expressed in endothelial cells (eNOS), in inhibitory non-adrenergic noncholinergic nerves (nNOS), and epithelial cells (nNOS and eNOS) [38]. However, in the present study we did not evaluate the expression of constitutive nitric oxide enzymes to confirm this possibility.

Interestingly, the animals that were also treated with nor-NOHA showed a lower amount of iNOS positive cells in lung tissue. Our hypothesis is that during the period of the experiment in which the distal lung slices were in contact with nor-NOHA, there was a decrease in NF-kB expression with a subsequent decrease in both arginase 2 and iNOS expression.

To test this hypothesis we evaluated the expression of NF-kB in distal lung tissue from the five experimental groups. We demonstrated that both treatments with 1400 W and NOR-NOHA diminished the NF-kB expression in sensitized animals. However, the association of 1400 W and nor-NOHA did not modify this response in sensitized animals. In this sense, Ckless et al. [14] also showed that inhibition or knockdown of arginase caused a small but consistent attenuation of basal and TNF-α stimulated NF-kB transcriptional activity. We also used a specific iNOS inhibitor (1400 W). In addition, we previously had shown that L-NAME effects were mostly due to constitutive NOS blockade and different forms specific of iNOS inhibitions [6].

NF-kB is an essential transcription factor not only for the induction of iNOS, but also for the up-regulation of CAT-2B. The simultaneous up-regulation of CAT-2B with iNOS is considered as a mechanism to ensure a high substrate supply for iNOS. Hammermann et al. (2000) [44] clearly showed that these results were evaluated after 20 hs of culture contact with L-arginine. In addition, we clarified that in the present study. The animals received 1400 W (iNOS inhibitor) daily during 72 hs before the experiment. Then, this time was sufficient to inhibit NF-KB expression.

We also showed that there was a reduction of 8-isoprostane expression in animals exposed to ovalbumin that received either nor-NOHA or 1400 W treatment. Ogino et al. [45] showed that arginase 1 protein expression in blood serum has recently been associated with PGF2α in healthy Japanese people and may become a new biomarker for the early prediction of oxidative stress-related diseases. These findings also corroborate the idea that not only iNOS but also arginase activation increases activation of the oxidative stress pathway. As previously shown, this may have detrimental effects on lung tissue structure [2,3].

Another point to be analyzed is that the association of both inhibitors caused larger reductions than either treatment alone in isoprostane PGF2α expression. This was sufficient to reduce PGF2α but did not affect the distal lung mechanical responses. Considering the importance of isoprostanes as contractile agents, we expected that there was a more prominent effect on lung mechanics in animals that received the two inhibitors. In addition, De Boer et al. [46] showed that the increased formation of the highly reactive procontractile and proinflammatory oxidant peroxynitrite plays an important role in airway hyperresponsiveness after the late airway responsiveness. It is possible that differences in the protocols of treatment with these inhibitors and/or different doses may contribute to attenuate the mechanical responses observed. However, this needs to be tested in another study.

Reinforcing the previous results, we showed that the resistance of the distal lung was correlated with the number of iNOS positive cells, isoprostane PGF2α and NF-kB expression [28,47]. In the present study we also showed that arginase 2 was correlated to resistance of distal lung tissue. Another point is that the NF-kB expression was correlated to arginase 2 expression and the number of iNOS positive cells. These results suggested a mechanistic pathway that needs to be further investigated.

Although we had previously mentioned some limitations of the present study, we also note that the results obtained in animal models may not be always applicable in humans. Nevertheless, it is important to remember that the guinea pig model is one of the best animal models to study asthma, as its lung anatomy is similar to humans, and it mimics some of the characteristics of human asthma such as the eosinophilic inflammatory and remodeling process [4,48]. Another point is that we evaluated the distal lung parenchyma. In fact, more studies need to be performed using chronic treatment with arginase inhibitors in intact animals. In the present study, the main limitation for performing this systemic treatment is the high cost of the drug. Finally, we also need to evaluate not only the expression of iNOS but also the activity of this enzyme, as well as the evaluation of constitutive nitric oxide sintase isoforms.

## Conclusions

In conclusion, this present study demonstrated that increased arginase 2 expression and activity contributes to increased iNOS expression and for the constriction of the distal lung parenchyma, likely due to altered NF-kB expression. These alterations associated with high PGF2α expression. Treatment with iNOS and arginase specific inhibitors reduced 8-isoprostane expression. The modulation of these pro-inflammatory pathways may represent future pharmacological tools for controlling pulmonary functional alterations induced by chronic inflammation.

## Abbreviations

AW: Alveolar wall; BVW: Blood vessel wall; BW: Bronchial wall; cNOS: Nitric oxide synthase constitutive; eNOS: Endothelial nitric oxide; Et: Tissue elastance; iNOS: Inducible nitric oxide synthase; L-NAME: NG-nitro-L-arginine methyl ester; NF-Kb: Nuclear factor-Kb; nor-NOHA: Nω-hydroxy-nor-Larginine; NO: Nitric oxide; NOS: Nitric oxide synthases; RNS: Reactive nitrogen species; ROS: Reactive oxygen species; Rt: Tissue resistance; 1400W: N-(3(Aminomethyl) Benzyl) Acetamidine; FAPESP: Foundation for Research Support of the State of São Paulo; LIM-20: Laboratory of Medical Investigation; HC: Hospital and Clinics; FMUSP: Faculty of Medicine, University of São Paulo.

## Competing interests

The authors declare that they have no competing interests.

## Authors’ contributions

LRCRBA acquired the data, analyzed and interpreted the data, contributed to design of the study, and drafted and revised the manuscript. RFR, NM, RBF, CMS, JCPS, PA and contributed to acquiring the data, interpretation of data, and drafted parts of the manuscript and made revisions to the manuscript. EAL, LCC, CMP, MD, MAM and IFLCT made the design of the study, contributed to the interpretation of data, and drafted parts of the manuscript and made critical revisions to the manuscript. All authors read and approved the final manuscript.

## Pre-publication history

The pre-publication history for this paper can be accessed here:

http://www.biomedcentral.com/1471-2466/13/52/prepub
